# Identification of Floral Volatile Components and Expression Analysis of Controlling Gene in *Paeonia ostii* ‘Fengdan’ under Different Cultivation Conditions

**DOI:** 10.3390/plants12132453

**Published:** 2023-06-26

**Authors:** Huili Ma, Chenjie Zhang, Tongfei Niu, Meida Chen, Lili Guo, Xiaogai Hou

**Affiliations:** 1 College of Horticulture and Plant Protection, Henan University of Science and Technology, Luoyang 471023, China; xiaoma_77@163.com; 2 College of Agriculture/Tree Peony, Henan University of Science and Technology, Luoyang 471023, China; zhangchenjie@stu.haust.edu.cn (C.Z.); niutongfei@stu.huast.edu.cn (T.N.);

**Keywords:** *Paeonia ostii*, different cultivation conditions, floral volatile components, controlling gene

## Abstract

In order to explore the release rule of floral volatile substances and the diurnal variation of different flower development stages of *Paeonia ostii* ‘Fengdan’ in potted and ground-planted conditions, dynamic headspace adsorption combined with gas chromatography-mass spectrometry(GC-MS) was used to analyze the dynamic changes in floral volatile components and contents. Quantitative real-time PCR (qRT-PCR) was used to analyze changes in flower fragrance-regulating genes *PsPAL*, *PsTPSs*, and *PsbHLH* at different flower development stages and a daily change process at the full-blooming stage. The results show that there were differences in aroma components and contents of *Paeonia ostii* ‘Fengdan’ at different flower development stages and different time quantum of every day. There were 25 and 28 aroma components identified in 7 flower development stages of tree peonies planted in pots and in the field, respectively, and 23 and 22 aroma components identified at different time quantum of the day, of which the largest and highest content was alkanes. The main characteristic aroma substances were (E)-β-ocimene, 1,3,5-trimethoxybenzene, 2,4-di-tert-butylphenol, methyl jasmonate, nerol, and cinnamyl alcohol; released amounts of the abovementioned substances varied depending on the development stage and the time of the day. The expression of flower fragrance-controlling genes (*PsPAL*, *PsTPSs*, and *PsbHLH*) in tree peonies varied greatly in different conditions. The results of this study provide a valuable resource to investigate floral fragrance formation in tree peonies.

## 1. Introduction

The tree peony (*Paeonia suffruticosa* Andr.) is a candidate for the national flower of China; its flowers are big, colorful, elegant, and dignified [1]. The cultivation of tree peonies has had a long history and uses a variety of systems. Tree peonies have nine flower colors and ten flower types, which have been formed after a long stage of manual selection. The flower color, flower type, flowering time, flower fragrance, and oil of the tree peony have an important economic value. There is relatively extensive research on properties of flower color [2], flower type [3], flowering time [4,5,6], and oil [7], while the research on flower fragrance is still in the initial stages. The study on the formation of flower fragrance and its regulation mechanism in tree peonies is of great significance.

Floral fragrance, a mixture of secondary metabolites released from flower and vegetative organs of plants, is composed of low-molecular-weight volatile components [8]. Each floral volatile component has individual specificity, whose type, content, and proportion is different in different kinds of plants [9]. The floral aroma components are also diversified. The amount of fragrance released varies at different development stages and tissues and organs of the flower [10]. So far, more than 1700 kinds of floral volatile components have been identified from more than 1000 species of flowers in 90 genera, which are mainly divided into three categories, including terpenoids, phenylpropanoid/benzenoid and fatty acid derivatives, and amino acid derivatives [11]. Among them, terpenoids and phenylpropanoids/benzenoids contribute a lot to the fragrance of flowers and are the main components of most plants [12]. Flower fragrance plays an important role in promoting the development of agriculture, biology, medicine, cosmetics, spices, essence, and other industries [13]. With in-depth research on the influencing factors of floral aroma components and the regulatory mechanisms of floral aroma genes, the metabolic pathways of plant floral aroma have gradually been revealed.

Dozens or even hundreds of aromatic substances with different contents could be released from tree peony flowers—mainly terpenes, alcohols, benzene ring/phenylpropanoids, and alkanes, but also slight esters, aldehydes, ethers, and other compounds [14]. Li et al. [15] divided 30 cultivated varieties into 5 flavor types: woody, rose, lily of the valley, phenolic, and unknown flavor, among which cis ocimene, d-citronellol, linalool, 1,3,5-trimethoxybenzene, and pentadecane are considered the main floral fragrances. A total of 124 volatile components were identified from 9 wild species of the tree peony, including 5 kinds of terpenoids, alkanes, alcohols, aldehydes, and ketones [16]. The research on the edible tree peony variety *Paeonia ostii* ‘Fengdan’ mainly focuses on the optimization of the seed oil extraction process, fatty acid composition analysis, and planting mode optimization; however, the research on flower fragrance is still insufficient.

The research on floral fragrance-controlling genes has just started, mainly focusing on genetic engineering-related research, such as floral fragrance gene cloning. Terpenoid Synthase (TPS) is a key enzyme for the synthesis of terpenoids, and the *TPSs* gene family has the function of catalyzing the synthesis of aromatic substances, such as linalool and nerolidol tertiary alcohol [17]. Phenylalanine ammonia lyase (PAL) expression is regulated by phenylpropanoid metabolites [18]. The transcription and accumulation of the *PAL* gene in the tobacco flower maturation process showed a downward trend during flower development, indicating that *PAL* had a certain correlation with flower opening and flower fragrance formation [19]. *bHLHs* often coordinate with other transcription factors to regulate the biosynthesis of secondary metabolites, such as terpenoids, phenylpropanoids, etc. Aslam screened two *bHLH* family transcription factors (*CpMYC2* and *CpbHLH13*) from the transcriptome library constructed at different flower development stages of *Chimonanthus praecox*. Overexpression of these two transcription factors in *Arabidopsis* and tobacco revealed that they both promoted monoterpene (linalool) and sesquiterpene (β-caryophyllene), indicating that they play an important role in the synthesis of volatile substances during flowering [20].

Dynamic headspace adsorption combined with gas chromatography-mass spectrometry (GC-MS) was used to study the characteristics of floral volatile components and content (percentage of each substance in the total substance content) changes in *Paeonia ostii* ‘Fengdan’ at different flower development stages under pot and field-planted conditions and study the diurnal variation of the volatile components of the tree peony at the full-blooming stage. At the same time, qRT-PCR was used to identify the spatiotemporal expression pattern of flower fragrance-regulating genes. This study provides a theoretical basis for cultivating and improving tree peony varieties and developing tree peony essential oil and other products.

## 2. Results

### 2.1. Analysis of Flower Volatile Components at Different Flower Development Stages of Potted Paeonia ostii ‘Fengdan’

The volatile components of seven different flower development stages of potted *Paeonia ostii* ‘Fengdan’ were detected by GC-MS, and the results are shown in Table S1. A total of 25 volatile components were detected, which were divided into 6 categories of alcohols, hydrocarbons, esters, terpenes, benzene rings, and phenols. Hydrocarbons were the most abundant, with eleven species, but phenol only had one type. There were two kinds of alcohols, four kinds of esters, three kinds of terpenes, and four kinds of benzene ring compounds.

During the seven flower development stages, 17 compounds were detected at the color exposure stage, including 8 hydrocarbons, 4 benzene rings, 3 esters, 1 terpene, and 1 phenol, respectively, with no alcohol detected. At the blooming stage, 20 compounds were detected in the sample. Hydrocarbons, benzene rings, and ester compounds were the main substances at this stage. Geraniol, (z)-7-tetradecen-1-yl acetate, phytane, 3-methylheptadecane, and nonadec-1-ene were not detected, and (E)-β-ocimene and 1,3,5-trimethoxybenzene were the two compounds with the highest content at this stage, 2.943 µg g^−1^ and 2.895 µg g^−1^, respectively. At the initial flowering stage, 10 hydrocarbon compounds—4 benzene rings, 2 terpenes, 2 alcohols, 2 esters, and 1 phenol—were detected, of which 1,3,5-trimethoxybenzene had the highest content, whereas (3Z)-hex-3-en-1-yl acetate, methyl jasmonate, phytane, and nonadec-1-ene were not detected in the samples at this stage. At the half-opening stage, 9 hydrocarbons, 3 benzene rings, 3 esters, 2 alcohols, and 2 terpenes were detected, with a total of 19 compounds. The content of (E)-β-ocimene was the highest (6.486 µg g^−1^), followed by 1,3,5-trimethoxybenzene and pentadecane. At the full-blooming stage, 10 hydrocarbons, 4 benzene rings, 2 alcohols, 2 terpenes, and 2 esters were detected, among which the content of (E)-β-ocimene was the highest, followed by 1,3,5-trimethoxybenzene and geraniol. A total of 21 substances were detected at the initial decay stage—10 hydrocarbons, 4 benzene rings, 2 alcohols, 2 terpenes, 2 esters, and 1 phenol—and the content of geraniol was the highest (1.179 µg g^−1^). Compounds detected at the decay stage included 11 hydrocarbons, 4 benzene rings, 3 terpenes, 3 esters, 2 alcohols, and 1 phenol. The content of geraniol was the highest, reaching 3.385 µg g^−1^.

### 2.2. Analysis of Flower Volatile Components at Different Flower Development Stages of Field-Planted Paeonia ostii ‘Fengdan’

After identification and calculation, the volatile components and contents of the field-planted *Paeonia ostii* ‘Fengdan’ at different flower development stages are shown in Table S2. The results show that 28 compounds were detected in the samples from different flower development stages; these compounds could be divided into hydrocarbons, alcohols, terpenes, benzene rings, esters, phenols, and aldehydes.

At the color exposure stage, 22 compounds were detected in the sample, most of which were hydrocarbons, terpenes, and alcohols; 1,3,5-trimethoxybenzene was the most abundant substance at this stage, with the content reaching 4.407 µg g^−1^; however, undecane, phenylethyl alcohol, 3-phenylpropanol, nerol, cinnamyl alcohol, and (+)-3-carene were not detected. There were 8 hydrocarbon compounds, 5 terpenes, 5 alcohols, 2 benzene rings and 1 phenol at the blooming stage; the content of (E)-β-ocimene was the highest, 4.319 µg g^−1^; heptane, 2,2,4,6,6-pentamethyl-, (3Z)-hex-3-en-1-yl acetate, undecane, nonanal, phenylethyl alcohol, (−)-myrtenol, decanal, and nonadec-1-ene were not detected in the samples of this stage. At the initial flowering stage, 24 substances were detected, including 9 hydrocarbons, 6 terpenes, 5 alcohols, 2 benzene rings, 1 ester and 1 phenol. (E)-β-ocimene had the highest content (5.002 µg g^−1^), but heptane, 2,2,4,6,6-pentamethyl-, (−)-myrtenol, decanal and 3-phenylpropanol were not detected. Only 11 volatile components were detected at the half-opening stage, including 5 hydrocarbons, 2 alcohols, 2 terpenes, 1 ester and 1 benzene ring. Among them, pentadecane, heptadecane, and phenylethyl alcohol had higher content. At the full-blooming stage, 8 hydrocarbons, 3 alcohols, 2 terpenes and 1 benzene ring were detected, of which pentadecane had the highest content, followed by 1,3,5-trimethoxybenzene. A total of 14 compounds—7 hydrocarbons, 3 terpenes, 2 alcohols, and 2 benzene rings—were detected at the initial decay stage, with the highest content of pentadecane. The substances detected at the decay stage had the lowest content, with only 10 species, including hydrocarbons, terpenes, and benzene rings. In general, the most volatile compounds were detected at the initial flowering stage, and the lowest content was detected at the decay stage.

### 2.3. Analysis of the Content of Flower Volatile Components in Different Flower Development Stages of Potted and Field-Planted Paeonia ostii ‘Fengdan’

There are 16 kinds of common components in potted and field-planted tree peonies, mainly hydrocarbon compounds. There are 12 kinds of unique volatile components in potted tree peonies, which are styrene, 3-hexanol, heptane, 2,2,4,6,6-pentamethyl-, nonanal, phenylethyl alcohol, (−)-myrtenol, decanal, 3-carene, 3-phenylpropanol, cinnamyl alcohol, (+)-3-carene and 1-octadecene. However, ethylbenzene, p-xylene, hexyl methacrylate, geraniol, methyl jasmonate, (z)-7-tetradecen-1-yl acetate, 8-heptadecene, phytane, and 3-methylheptadecane were only found in the field-planted tree peonies.

**Figure 1:**
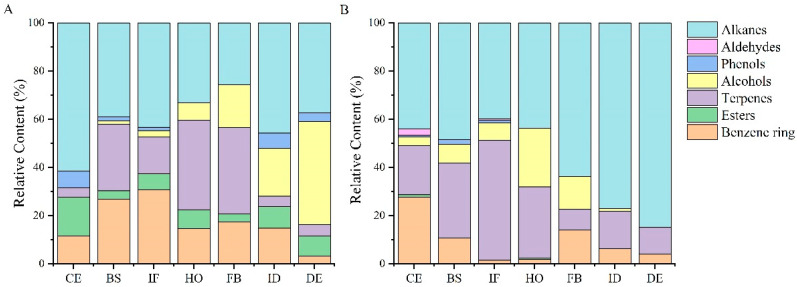
Classification and content of volatile components at different flower development stages of potted and field-planted *Paeonia ostii* ‘Fengdan’. (**A**) potted *Paeonia ostii* ‘Fengdan’; (**B**) field-planted *Paeonia ostii* ‘Fengdan’; CE: color exposure stage; BS: blooming stage; IF: initial flowering stage; HO: half-opening stage; FB: full-blooming stage; ID: initial decay stage; DE: decay stage.

The contents of aroma substances of different types vary between flower development stages of *Paeonia ostii* ‘Fengdan’ under two cultivation conditions. Figure 1 shows that the content of benzene ring compounds is the highest at the initial flowering stage of potted *Paeonia ostii* ‘Fengdan’, whereas the field-planted *Paeonia ostii* ‘Fengdan’ has the highest content at the color exposure stage. The content of ester compounds in potted and field-planted *Paeonia ostii* ‘Fengdan’is the highest at the color exposure stage, but these compounds are not present at the flowering, full-blooming, initial decay, and decay stages in a field-planted *Paeonia ostii* ‘Fengdan’. The content of terpenes is the highest in the half-opening stage and full-blooming stage of potted *Paeonia ostii* ‘Fengdan’, and it is also the highest at the initial flowering stage of the field-planted *Paeonia ostii* ‘Fengdan’. Alcohols gradually increase during the seven development stages of potted plants and reach the highest level at the decay stage; however, they increase first and then decrease in field-planted *Paeonia ostii* ‘Fengdan’ and reach the highest level at the half-opening stage. The content of phenols is low, and they do not appear at half-opening and full-blooming stages of the tree peony under the two cultivation conditions. Aldehydes appear only at the color exposure and initial flowering stages of the field-planted *Paeonia ostii* ‘Fengdan’. Hydrocarbons are the most abundant substances at the color exposure, blooming, initial flowering, and initial decay stages of potted plants, with the highest content occurring at the color exposure stage. Terpenes are the most important floral substances at the half-opening and full-blooming stages, while alcohols are the most important floral substances at the decay stage. In addition to the highest content of terpenes at the initial flowering stage, alkanes are the most important substances at each stage of the field-planted tree peonies.

### 2.4. Analysis of Characteristic Aroma at Different Flower Development Stages

**Figure 2:**
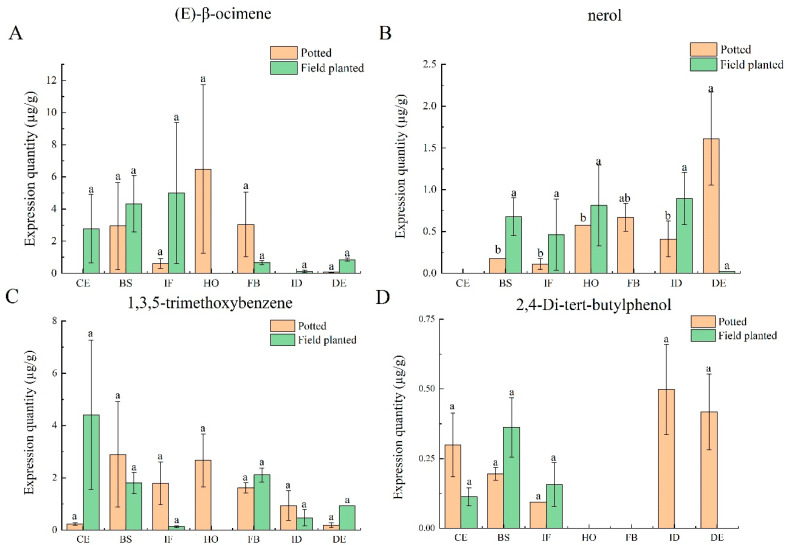
Characteristic aroma analysis of potted and field-planted *Paeonia ostii* ‘Fengdan’ at different flower development stages. CE: color-exposure stage; BS: blooming stage; IF: initial flowering stage; HO: half-opening stage; FB: full-blooming stage; ID: initial decay stage; DE: decay stage. Different lowercase letters represent significant differences, *p* < 0.05. Error bars, standard error of the mean (SEM). (**A**) The expression quantity of (E)-β-ocimene in different flower development stages under two cultivation conditions; (**B**) The expression quantity of nerol in different flower development stages under two cultivation conditions; (**C**) The expression quantity of 1,3,5-trimethoxybenzene in different flower development stages under two cultivation conditions; (**D**) The expression quantity of 2,4-di-tert-butylphenol in different flower development stages under two cultivation conditions.

The characteristic aroma substances common to the seven flower development stages under two cultivation conditions include (E)-β-ocimene, nerol, 1,3,5-trimethoxybenzene, and 2,4-di-tert-butylphenol. The results show that the release amounts of four characteristic aroma substances were different at the seven flower development stages under different cultivation conditions. (E)-β-ocimene appeared in the blooming stage, initial flowering stage, half-opening stage, full-blooming stage, and decay stage of potted *Paeonia ostii* ‘Fengdan’, and the release amount is shown as follows: half-opening stage > full-blooming stage > blooming stage > initial flowering stage > decay stage. The release amount of ‘Fengdan’ planted in the field is as follows: the initial flowering stage > blooming stage > color exposure stage > decay stage > full-blooming stage > initial decay stage (Figure 2A). The release amount of nerol at the decay stage of potted *Paeonia ostii* ‘Fengdan’ is significantly different from that at the blooming, initial flowering, half-opening, and initial decay stages (*p* < 0.05). There is no significant difference in the field-planted *Paeonia ostii* ‘Fengdan’ (Figure 2B). 1,3,5-trimethoxybenzene was detected at the seven stages of the potted tree peony, and the highest release amount occurred at the blooming stage. It was not detected at the half-opening stage of plants in the field, and the release amount was the highest at the color exposure stage (Figure 2C). 2,4-di-tert-butylphenol released at the initial decay stage of the potted plant was significantly higher than that at other stages; the release of 2,4-di-tert-butylphenol was only detected at the color exposure, flowering, and initial flowering stages in the field-planted *Paeonia ostii* ‘Fengdan’ (Figure 2D).

### 2.5. Analysis of Daily Changes in Flower Volatile Components of Potted Paeonia ostii ‘Fengdan’

Daily changes in volatile components of potted *Paeonia ostii* ‘Fengdan’ flowers were obtained by GC-MS (Table S3). A total of 23 substances were detected at four time stages, including 12 hydrocarbons, 4 benzene rings, 3 terpenes, 2 esters, 1 phenol, and 1 aldehyde.

A total of 18 volatile components were collected from 6:00 to 9:00 a.m., including 10 hydrocarbons, 4 benzene rings, 2 esters, 1 terpene, and 1 phenol. From 9:00 to 12:00, 14 substances were detected, including 6 hydrocarbons, 3 benzene rings, 2 esters, 2 terpenes, and 1 phenol. Ten volatile components and twenty-two volatile components were detected, respectively, during the hours of 12:00–15:00 and 15:00–18:00. Decane, phytane, and nonadec-1-ene were not detected in the sample collected from 12:00 to 15:00, and methyl jasmonate was not detected in the sample collected from 15:00 to 18:00. The substance with the highest content detected during four-time stages was 1,3,5-trimethoxybenzene.

### 2.6. Analysis of Daily Change of Flower Volatile Components of Field-Planted Paeonia ostii ‘Fengdan’

As shown in Table S4, we can divide twenty-two substances detected in the daily changes of volatile components in the field-planted tree peony into five categories: hydrocarbons, terpenes, alcohols, benzene rings, and phenols. Among them, hydrocarbons are heptane, 2,2,4,6,6-pentamethyl-, hexane, 3,3-dimethyl-, undecane, dodecane, tridecane, tetradecane, pentadecane, hexadecane, 2-methylhexadecane, heptadecane, octadecane, and nonadecane,2-methyl-. There are four kinds of terpenes, namely, (E)-β-ocimene, 3-carene, nonadec-1-ene and 1-eicosene. There are three kinds of alcohols, namely 3-hexanol, nerol, and cinnamyl alcohol. There are two kinds of benzene rings, m-xylene and 1,3,5-trimethoxybenzene. There is one kind of phenol, 2,4-di-tert-butylphenol.

Twenty-two volatile components were collected from 6:00 to 9:00 and from 9:00 to 12:00. Heptane, 2,2,4,6,6-pentamethyl-, hexane,3,3-dimethyl- and nonadecane,2-methyl- were not detected in the sample collected from 12:00 to 15:00. Dodecane and nonadecane,2-methyl- were not detected in the sample collected from 15:00 to 18:00.

### 2.7. Classification and Content Analysis of Daily Change of Flower Volatile Components

**Figure 3:**
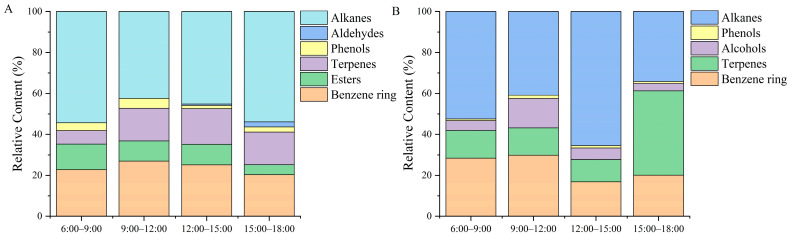
Classification and content of daily volatile components in potted and field-planted *Paeonia ostia* ‘Fengdan’. (**A**) potted *Paeonia ostia* ‘Fengdan’; (**B**) field-planted *Paeonia ostia* ‘Fengdan’.

An initially increasing and then decreasing trend appeared in benzyclic substances of potted *Paeonia ostii* ‘Fengdan’ at different time stages of the day, and the content was the highest from 9:00 to 12:00 (Figure 3A). The content of benzene ring substances in ground-planted *Paeonia ostii* ‘Fengdan’ was also the highest from 9:00 to 12:00 (Figure 3B). Esters were the highest during the hours of 6:00–9:00 and were only detected in potted tree peonies. Terpenes showed a trend of first increasing and then decreasing levels in potted *Paeonia ostii* ‘Fengdan’, with the largest amount released from 12:00 to 15:00. However, terpenes first decreased and then increased in field-planted *Paeonia ostia* ‘Fengdan’, and the lowest content was from 12:00 to 15:00. Alcohols were only contained in the field-planted *Paeonia ostii* ‘Fengdan’, and the release amount from 9:00 to 12:00 was significantly higher than that at other time stages. Phenols and aldehydes were relatively low compared with other compounds, and their release amount was significant from 9:00 to 12:00. Alkanes were the compounds with the highest content under the two cultivation conditions, showing a trend of first decreasing and then increasing levels in potted tree peonies, with the lowest content from 9:00 to 12:00. The highest content was found from 12:00 to 15:00 under the field conditions, and the lowest content was detected from 15:00 to 18:00.

### 2.8. Characteristic Aroma Analysis of Daily Change in Potted and Field-Planted Paeonia ostii ‘Fengdan’ at Different Time Stages

**Figure 4:**
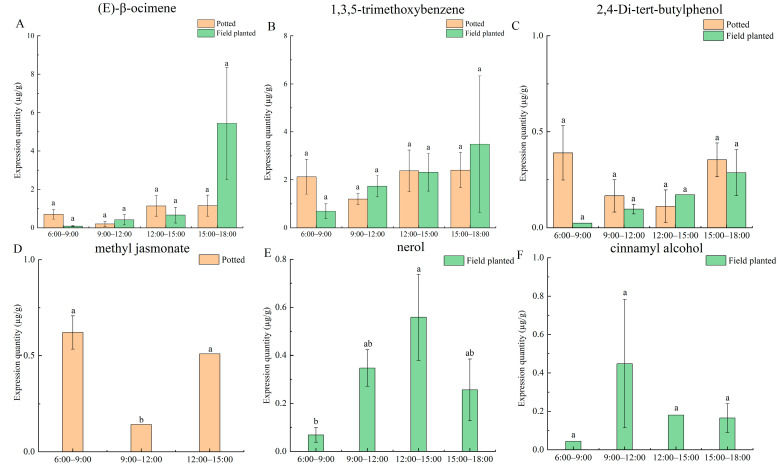
Daily change analysis of characteristic aroma of potted and field-planted *Paeonia ostii* ‘Fengdan’. Different lowercase letters represent significant differences, *p* < 0.05. Error bars, standard error of the mean (SEM). (**A**) The expression quantity of (E)-β-ocimene in different time stages under two cultivation conditions; (**B**) The expression quantity of 1,3,5-trimethoxybenzene in different time stages under two cultivation conditions; (**C**) The expression quantity of 2,4-di-tert-butylphenol in different time stages under two cultivation conditions; (**D**) The expression quantity of methyl jasmonate in different time stages under two cultivation conditions; (**E**) The expression quantity of nerol in different time stages under two cultivation conditions; (**F**) The expression quantity of cinnamyl alcohol in different time stages under two cultivation conditions.

The characteristic aroma substances detected in the daily change process under the two cultivation conditions include (E)-β-ocimene, 1,3,5-trimethoxybenzene, 2,4-di-tert-butylphenol, methyl jasmonate, nerol, and cinnamyl alcohol (Figure 4). It turns out the (E)-β-ocimene, 1,3,5-trimethoxybenzene, and 2,4-di-tert-butylphenol were expressed in potted and field-planted *Paeonia ostii* ‘Fengdan’. The highest release of (E)-β-ocimene was found in potted *Paeonia ostii* ‘Fengdan’ from 12:00 to 15:00, and in the field-planted *Paeonia ostii* ‘Fengdan’, from 15:00 to 18:00 (Figure 4A). The release amount of 1,3,5-trimethoxybenzene of potted *Paeonia ostii* ‘Fengdan’ showed no significant difference between four time points, which may be related to the stability of the temperature in the greenhouse. The release amount of the field-planted *Paeonia ostii* ‘Fengdan’ was the highest from 15:00 to 18:00 (Figure 4B). The highest release amount of 2,4-di-tert-butylphenol was from 6:00 to 9:00 in potted *Paeonia ostii* ‘Fengdan’, and in the field-planted ‘Fengdan’, from 15:00 to 18:00 (Figure 4C). Methyl jasmonate was only detected in potted *Paeonia ostii* ‘Fengdan’ and only at three-time stages, with the highest release from 6:00 to 9:00 (Figure 4D). The release amount of nerol and cinnamyl alcohol in the field-planted *Paeonia ostii* ‘Fengdan’ was the highest between 12:00 and 15:00 and between 9:00 and 12:00, respectively (Figure 4E,F).

### 2.9. Study on the Expression of Genes Related to Floral Fragrance Regulation

**Figure 5:**
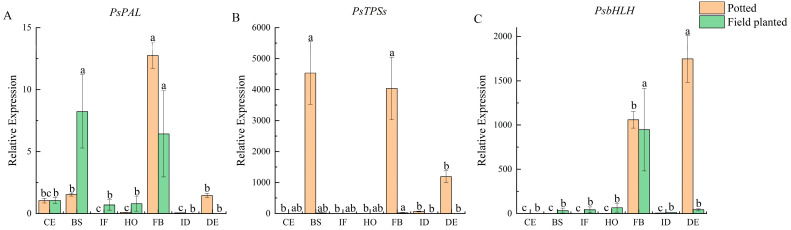
Expression analysis of genes related to floral fragrance regulation at different flower development stages. Bars represent the relative expression, determined by qRT-PCR, of *PsPAL*, *PsTPSs*, and *PsbHLH* at the seven flower development stages (CE: color exposure stage; BS: blooming stage; IF: initial flowering stage; HO: half-opening stage; FB: full-blooming stage; ID: initial decay stage; DE: decay stage) of potted (orange color) and field-planted (green color) *Paeonia ostii* ‘Fengdan’. Different lowercase letters represent significant differences, *p* < 0.05. Error bars, standard error of the mean (SEM). (**A**) Relative expression of *PsPAL* at the seven flower development stages; (**B**) Relative expression of *PsTPSs* at the seven flower development stages; (**C**) Relative expression of *PsbHLH* at the seven flower development stages.

The expression patterns of flower fragrance-regulating genes *PsPAL*, *PsTPSs*, and *PsbHLH* were investigated by qRT-PCR. During the seven flower development stages of potted and field-planted *Paeonia ostii* ‘Fengdan’, the relative expression level of *PsPAL* was the highest at the full-blooming stage, followed by the blooming stage (Figure 5A). The expression of the *PsTPSs* gene was significantly higher under potted conditions; it was also significantly higher at the blooming and full-blooming stages than at other stages (*p* < 0.05) (Figure 5B). The relative expression of the *PsbHLH* gene was the highest at the decay stage (*p* < 0.05), followed by the full-blooming stage. The relative expression at the full-blooming stage was the highest under field-planted conditions, which was significantly different from that at other stages (*p* < 0.05) (Figure 5C).

**Figure 6:**
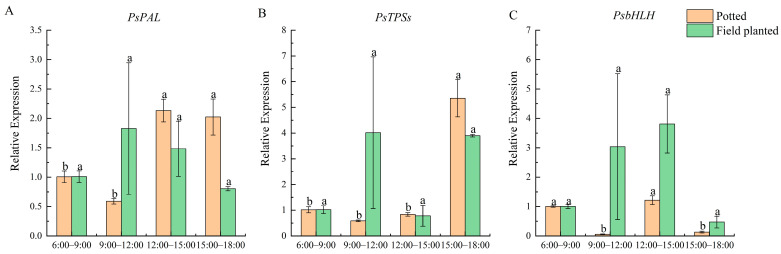
Expression analysis of the daily change of genes related to floral fragrance regulation. Bars represent the relative expression, determined by qRT-PCR, of *PsPAL*, *PsTPSs*, and *PsbHLH* at different times of the day for potted (orange color) and field-planted (green color) ‘Fengdan’. Different lowercase letters represent significant differences, *p* < 0.05. Error bars, standard error of the mean (SEM). (**A**) Relative expression of *PsPAL* at different times of the day; (**B**) Relative expression of *PsTPSs* at different times of the day; (**C**) Relative expression of *PsbHLH* at different times of the day.

At different time stages of the day, the expression of *PsPAL* was the highest from 12:00 to 15:00 and from 15:00 to 18:00, which was significantly higher than that at other stages (*p* < 0.05) for potted *Paeonia ostii* ‘Fengdan’. *PsPAL* did not change significantly throughout the day for the field-planted *Paeonia ostii* ‘Fengdan’ (Figure 6A). The expression of *PsTPSs* in potted tree peonies was the highest from 15:00 to 18:00, and the lowest from 9:00 to 12:00. The difference between the field- and the pot-planted peonies was detected from 9:00 to 12:00 (Figure 6B). The expression of *PsbHLH* was the highest from 12:00 to 15:00 under the two cultivation conditions, and the relative expression in potted *Paeonia ostii* ‘Fengdan’ was lower than that in the field (Figure 6C).

## 3. Discussion and Conclusions

The floral fragrance is one of the important indexes used to evaluate the quality of ornamental plants. People can smell different odors due to different types and amounts of volatile components released by plants under different conditions [21]. In this study, we analyzed the volatile components of *Paeonia ostii* ‘Fengdan’ from seven flower development stages and different time stages of the day under potted and field-planted conditions. Seven volatile components were identified, including alcohols, alkanes, esters, terpenes, benzene rings, phenols, and aldehydes. Of these, the main components are (E)-β-ocimene, 1,3,5-trimethoxybenzene, and geraniol, which is consistent with the previous research results [16]. Alkanes are the main volatile components of tree peonies, and the same conclusion was reached in the identification of floral components in F_1_ populations of *Paeonia ostii* ‘Fengdan’ and *Paeonia suffruticosa* ‘Chunguihuawu’ cross [22]. Zhang et al. [23] also found that the contents of hydrocarbons were relatively higher in the study of volatile components of tree peony varieties, such as *Paeonia suffruticosa* ‘Lvxiangqiu’ and *Paeonia suffruticosa* ‘Shouanhong’. N-alkane is one of the main components of plant leaf wax. Besides appearing on leaves, plant wax also appears on the surface of flowers or fruits of plants. Therefore, it is speculated that the flower surface of *Paeonia ostii* ‘Fengdan’ may be covered with a wax layer. In addition, terpenes, benzene rings, and alcohols are also volatile components with a high content found in this study, which is consistent with the results obtained by Li et al. [15] in the research of 30 tree peony varieties. 1,3,5-trimethoxybenzene is the most typical among benzene ring substances, which is consistent with the research results of Song et al. [24].

The content of volatile components is easily affected by the cultivation environment, flower development stage, and aroma collection method [25]. This study found that characteristic aroma substances were (E)-β-ocimene, nerol, geraniol, etc. at different cultivation conditions, different development stages, and different times of the day, which is consistent with the research results of Zhang et al. [23]. Changes occurred in volatile aroma compounds with daily rhythm, flowering stage, and different flower parts for *Oncidium.* Major aroma volatile compounds were linalool, benzaldehyde, β-myrcene, tiglaldehyde, benzyl alcohol, and nerolidol [26]. (E)-β-ocimene is a kind of acyclic monoterpene compound, which has a grass fragrance, flower fragrance, and orange flower oil smell. It is a common floral substance in many flowers and plants [27]. In the present study, the content of (E)-β-ocimene was the highest at the half-opening stage of potted *Paeonia ostii* ‘Fengdan’ and the initial flowering stage of the field-planted *Paeonia ostii* ‘Fengdan’. 1,3,5-trimethoxybenzene is a kind of benzene ring compound, which has a fresh and elegant fragrance [28] and 2,4-di-tert-butylphenol has a carbonaceous acid taste [29]. Methyl jasmonate widely exists in plants and exogenous spraying of methyl jasmonate can significantly promote the release of floral components of *Styrax japonicus* [30] and *Echinacea purpurea* L. Moench [31]. Nerol is one of the important components of flower aroma. Its release amount was the highest at the decay stage of potted *Paeonia ostii* ‘Fengdan’; however, there was no significant difference at the flower development stage of the field-planted *Paeonia ostii* ‘Fengdan’, but nerol’s content is affected by daily changes.

Terpenoid Synthase (TPS) is a key enzyme for the synthesis of terpenoids. Magnard et al. [32] identified the genes on the pathway related to the synthesis of terpenes and phenylpropanoid/benzene ring compounds from the genomic level and found that the *TPSs* gene family has the function of catalyzing the synthesis of aromatic substances, such as linalool, nerolidol, etc. In the study of volatile substances in *Jasminum sambac*, it was found that the release of linalool reached its peak after the opening of petals and was consistent with the maximum expression of the *JsTPS* gene, which may be related to the formation of jasmine fragrance [33]. Overexpression *TPS1* gene in *Magnolia champaca* indicated that *McTPS1* is linalool synthase, responsible for producing the most monoterpenes in cedar flowers [34]. Across tree peony cultivars, the expressions of three *TPS* genes *PdTPS1*, *PdTPS2* and *PdTPS4* were significantly positively correlated with linalool emissions [35]. Phenylalanine ammonia lyase (PAL) is the key rate-limiting enzyme of the phenylpropanoid metabolic pathway and is involved in regulating 1,3,5-trimethoxybenzone, 2,4-di-tert-butylphenol, and other benzene ring compounds. The expression of the *PAL* gene is regulated by the feedback of phenylpropanoid metabolic pathway products [36]. Jiang et al. [37] found that the expression of *LrPAL* in the *Lycoris radiata* flower was higher at the bud and withering stages than in the blooming stage. Aslam screened two bHLH family transcription factors (*CpMYC2* and *CpbHLH13*) from the transcriptome library of *Chimonanthus praecox* constructed at different flower development stages. Two transcription factors were the most expressed at the full-blooming stage, and overexpression of *CpMYC2* and *CpbHLH13* in *Arabidopsis* and tobacco both promoted the synthesis of monoterpene (linalool) and sesquiterpene (β-caryophyllene), indicating that they play an important role in the synthesis of volatile components at the flowering stage [20]. The specific regulatory mechanisms of these genes need to be studied further.

As one of the important indexes to evaluate the quality of tree peonies, the fragrance of different tree peonies has different intensity and characteristics due to the difference in aroma components and release amounts and has attracted more and more attention in recent years. In this study, 25 and 28 volatile components were identified during the seven flower development stages of potted and field-planted *Paeonia ostii* ‘Fengdan’, and 23 and 22 volatile components were identified at different time stages of a single day, with alkanes identified as the main components. The main aroma components were (E)-β-ocimene, 1,3,5-trimethoxybenzene, 2,4-di-tert-butylphenol, methyl jasmonate, nerol, and cinnamyl alcohol. The releasing trend of the main aroma components was different under different cultivation conditions and at different times of the day. The expression of floral fragrance-controlling genes in tree peonies varied greatly in different cultivation modes, at different development stages, and during daily changes. The relative expression level of *PsPAL* was the highest at the full-blooming stage, and the highest expression level was found in potted *Paeonia ostii* ‘Fengdan’ from 12:00 to 15:00 and from 15:00 to 18:00, while there was no significant difference in the field-planted *Paeonia ostii* ‘Fengdan’. *PsTPSs* gene was significantly higher at the blooming and full-blooming stages than in other stages. The highest expression level was found in potted *Paeonia ostii* ‘Fengdan’ from 15:00 to 18:00, while in the field-planted *Paeonia ostii* ‘Fengdan’, the highest expression level was found from 9:00 to 12:00. The relative expression of *PsbHLH* in potted plants was lower than that in the field, and the highest expression was from 12:00 to 15:00. Under different cultivation methods, the volatile components and contents of *Paeonia ostii* ‘Fengdan’ are different at different development stages and different time stages in a single day. This study provides a theoretical basis for revealing the formation and regulation mechanism of *Paeonia ostii* ‘Fengdan’ fragrance characteristics and also provides technical support for accelerating the industrial development and utilization of the tree peony fragrance.

## 4. Materials and Methods

### 4.1. Materials

Potted and field-planted *Paeonia ostii* ‘Fengdan’ were used as experimental materials in this study. On 1 November 2020, the plants were transplanted into the pots and were cultured in the greenhouse (17 ± 1 °C) for early flowering. The volatile components of potted *Paeonia ostii* ‘Fengdan’ were collected in the greenhouse (23 ± 1 °C) in January 2021. The potted containers were black plastic flowerpots with a height of 40 cm and a diameter of 35 cm. In April 2021, the volatile components of the flowers of *Paeonia ostii* ‘Fengdan’ planted in the same field were collected from the peony resource nursery of Luoyang Academy of Agriculture and Forestry Sciences. The fragrance of potted and field-planted *Paeonia ostii* ‘Fengdan’ was collected at different flower development stages (CE: color exposure stage; BS: blooming stage; IF: initial flowering stage; HO: half-opening stage; FB: full-blooming stage; ID: initial decay stage; DE: decay stage) and different time stages of the day (6:00–9:00, 9:00–12:00, 12:00–15:00 and 15:00–18:00) at the full-blooming stage, and the air around the tree peony was taken as a contrast. After collection, the adsorption tubes were sealed with fresh film and wrapped with tin foil, marked, and placed in a low-temperature ice box for storage in the refrigerator. Each treatment was repeated 5 times. Petal samples were collected at different flower development stages and different time stages of a single day, and three biological repeats were taken from each sample. The samples were immediately frozen in liquid nitrogen and stored at −80 °C until RNA extraction.

### 4.2. Collection of Volatile Components

The volatile components of potted and field-planted *Paeonia ostii* ‘Fengdan’ flowers were collected by dynamic headspace bagging adsorption collection method according to Li et al. [7]. In order to reduce the impact of environmental factors during the experiment, the sampling time for different flower development stages was set to start at 9:00 a.m. The flowers with the same size and opening degree were placed in the tasteless and transparent sampling bags with openings at both ends. The activated-carbon tube (outer diameter 6 mm, length 75 mm) was inserted at the upper end of the sampling bag, and the Tenax TA adsorption tube (outer diameter 6 mm, length 100 mm) was inserted at the lower end, and both ends were sealed with plastic strips. The odorless and transparent silica gel tube (5 × 7 mm) was used to connect the Tenax TA adsorption tube with the QC-1S air sampler (Beijing Institute of Labor Protection Sciences, Beijing, China). The uniform flow rate of the air sampler was 400 mL/min and the sampling time was 3 h. After sampling, 1000 μL n-hexane was added into the adsorption tube to get the eluent into a 2 mL brown injection bottle (Agilent, Santa Clara, CA, USA). Samples were stored in the refrigerator at −20 °C until they were taken out for instrument analysis.

### 4.3. Analysis Conditions of GC-MS

(1) GC conditions: The temperature at the injection port was 250 °C, and the injection volume was 3 μL. The carrier gas was high-purity helium (99.999%). The split injection method was adopted, and the flow rate was 1.0 mL/min. The capillary column was an elastic quartz capillary column HP-5MS (0.25 mm × 30 m, 0.25 μm). The heating program was as follows: the initial temperature was 70 °C; it was kept for 1 min and raised to 142 °C at the rate of 6 °C/min; then it was raised to 148 °C at the rate of 1 °C/min, and then raised again to 180 °C at the rate of 2 °C/min; finally, it was raised it to 250 °C at the rate of 10 °C/min and kept at that temperature for 20 min.

(2) MS conditions: The temperature of the ion source was 230 °C, the ionization source was EI, the ionization energy of EI was 70 eV, the temperature of the fourth pole was 260 °C, and the temperature of the transmission line was 250 °C. The scanning mode was full scanning, and the scanning range was 29–386 amu.

### 4.4. Qualitative and Quantitative Analysis of Volatile Components

The ethyl acetate solution with the concentration of 69.32 mg/L ethyl caproate was selected as the internal standard solution, and 0.4 μL of the solution was added to the 80 μL sample. GC-MS (Agilent 8890/6973A, USA) was used to detect the sample with added internal standard solution. The volatile components were separated by gas chromatography and identified by mass spectrometry, and the total ion current chromatograms consisting of chromatographic peaks of each component were obtained. The volatile components were identified through NIST 17 standard spectrum library retrieval and the related literature. The content of each component was calculated by using the peak area ratio of volatile matter to the internal standard. The following calculation formula was used:

Content of aroma substances (μg/g) = [peak area of each volatile substance/peak area of the internal standard] × Internal standard concentration (mg/L) × Internal standard volume (μL)/Sample weight (g) × F (f is the correction factor of each component to the internal standard, f = 1).

### 4.5. Analysis of Gene Expression Pattern of Floral Fragrance Regulation

Total RNA was extracted from collected petals using an RNAprep Pure Plant Kit (Polysaccharides and Polyphenolics-rich, TIANGEN). RNA quality and purity were confirmed by 1% agarose gel electrophoresis, using the DL2000 DNA Marker (TaKaRa, Dalian, China) as a size indicator. RNA concentrations and ratios of absorbance at 260 nm to that at 280 nm (260/280) were determined using a NanoDrop 1000 spectrophotometer (Implen, Munich, Germany). Total RNA was reverse transcribed into cDNA according to the steps of PrimeScript RT reagent Kit with gDNA Eraser (TaKaRa).

Based on the full-length transcriptome data of the third generation of the tree peony in the early laboratory, DNAMAN 8 and Primer Premier 5.0 were used to design specific amplification primers in the ORF frame sequence of candidate genes, and primers without primer dimer, hairpin structure, mismatch, and other information were selected. The primer sequence shown in Table 1 was synthesized by Bioengineering (Shanghai, China) Co., Ltd. *EF1-α* gene was used as an internal control to normalize gene expression.

### 4.6. Data Analysis

Excel 2013 and SPSS 21 software were used for statistical analysis, and Origin 2018 software was used for generating figures.

## Figures and Tables

**Table 1:** Primer sequences of real-time fluorescent quantitative PCR.

Gene Name	Forward-Primer Sequence	Reverse-Primer Sequence
*PsPAL*	TGCCTCGCTACTTACCCTTT	GGCTCTCGTATTCGCTTCTA
*PsTPSs*	ATGGCATTGCGTTTTTCTTTG	TGGCTGATCGCCTGTTTGTAG
*PsbHLH82*	CGTGAAAGGGTCAAATGGG	TCAGTTGGCGGACAAGTCG
*EF1-α*	CCGCCAGAGAGGCTGCTAAT	GCAATGTGGGAAGTGTGGCA

The TaKaRa TB Green Premium Ex Taq Ⅱ kit was used for qRT-PCR experiment, and the reaction system was as follows: 10 μL TB Green Premix Ex Taq Ⅱ, 0.8 μL For-primer and Rev-primer, respectively, 0.4 μL ROX Reference Dye Ⅱ, 6 μL RNase free water, and 2 μL cDNA solution. The cycle procedure was as follows: pre-denaturation 95 °C, 30 s; denaturation at 95 °C for 5 s; annealing and extension at 60 °C for 3 s, 39 cycles. Results were calculated using the formula of relative expression = 2^−ΔΔCt^.

## Data Availability

The datasets generated during and/or analyzed during the current study are listed in the text and its additional files.

## Supplementary Materials

The following supporting information can be downloaded at: https://www.mdpi.com/article/10.3390/plants12132453/s1, Tables S1 Analysis of floral volatile components of Paeonia ostia ‘Fengdan’ at different flower development stages under potted cultivation; Tables S2 Analysis of floral volatile components of Paeonia ostia ‘Fengdan’ at different flower development stages under field cultivation; Tables S3 Daily variation analysis of floral volatile components of Paeonia ostia ‘Fengdan’ under potted cultivation; Tables S4 Daily variation analysis of floral volatile components of Paeonia ostia ‘Fengdan’ under field cultivation.
