# Supplementary material for: Identification of Floral Volatile Components and Expression Analysis of Controlling Gene in Paeonia ostii ‘Fengdan’ under Different Cultivation Conditions
Source: Plants (Basel). 2023 Jun 26;12(13):2453. doi: 10.3390/plants12132453 (PMC10346646; doi:10.3390/plants12132453)
Supplement: Supplementary file 1 [file plants-12-02453-s001.zip › plants-2447251-supplementary.pdf]

Supplementary Material

Identification of Floral Volatile Components and Expression Analysis of Controlling Gene in *Paeonia ostii* 'Fengdan' under Different Cultivation Conditions

Huili Ma [1,†], Chenjie Zhang [2,†], Tongfei Niu [2], Meida Chen [2], Lili Guo [2,*] and Xiaogai Hou [2,*]

[1] College of Horticulture and Plant Protection, Henan University of Science and Technology, Luoyang 471023, China; xiaoma_77@163.com

[2] College of Agriculture/Tree Peony, Henan University of Science and Technology, Luoyang 471023, China; zhangchenjie@stu.haust.edu.cn (C.Z.); niutongfei@stu.huast.edu.cn (T.N.); d972749522@163.com (M.C.)

* Correspondence: guolili@haust.edu.cn (L.G.); hkdhxg@haust.edu.cn (X.H.); Tel.: +86-185-6760-5906 (L.G.); +86-0379-69980776 (X.H.)

† These authors contributed equally to this work.

Table S1 Analysis of floral volatile components of *Paeonia ostia* ‘Fengdan’ at different flower development stages under potted cultivation

Peak Time	Substance Name	CE	BS	IF	HO	FB	ID	DE
3.025	Ethylbenzene	0.049±0.001	0.094±0.020	0.049±0.013	0.042±0.009	0.054±0.013	0.055±0.006	0.042±0.006
3.112	p-Xylene	0.145±0.011	0.240±0.077	0.185±0.047	0.132±0.024	0.123±0.006	0.112±0.017	0.112±0.013
3.406	m-Xylene	0.069±0.009	0.124±0	0.062±0.014	/	0.041±0.006	0.068±0.020	0.042±0.005
4.171	Hexyl methacrylate	0.063±0.020	0.071±0.01	0.044±0.006	0.057±0.008	0.045±0.007	/	/
5.087	(3Z)-hex-3-en-1-yl acetate	0.232±0	0.113±0.063	/	0.591±0	/	/	0.101±0
5.897	(E)-β-ocimene	/	2.942±2.712	0.613±0.308	6.486±5.244	3.048±2.009	/	0.071±0.024
6.923	Undecane	0.084±0.036	0.140±0.052	0.075±0.036	0.122±0.056	0.054±0.003	0.107±0.013	0.064±0.015
9.075	Dodecane	0.174±0.043	0.171±0.071	0.118±0.061	0.167±0.095	0.084±0.001	0.128±0.040	0.113±0.022
9.718	nerol	/	0.177±0	0.113±0.065	0.576±0	0.668±0.168	0.411±0.216	1.611±0.556
10.274	geraniol	/	/	0.061±0	0.809±0.305	1.202±0.447	1.179±0.849	3.385±0.921
11.266	Tridecane	0.142±0.044	0.275±0.135	0.315±0.154	0.776±0.425	0.242±0.027	0.189±0.073	0.341±0.095
13.558	Tetradecane	0.329±0.053	0.298±0.102	0.204±0.067	0.401±0.099	0.149±0.039	0.298±0.032	0.307±0.048
13.846	1,3,5-trimethoxybenzene	0.235±0.050	2.895±2.014	1.789±0.818	2.671±1.010	1.620±0.202	0.943±0.563	0.192±0.086
16.480	Pentadecane	0.306±0.086	1.523±0.983	0.839±0.284	2.764±0.406	0.818±0.141	0.611±0.166	0.725±0.103
16.901	2,4-Di-tert-butylphenol	0.300±0.114	0.196±0.022	0.094±0	/	/	0.498±0.162	0.418±0.136
20.276	hexadecane	0.728±0.128	0.545±0.185	0.199±0.028	0.217±0.106	0.263±0.086	0.745±0.048	0.862±0.087
22.305	(-)-methyl jasmonate	0.395±0.197	0.257±0	/	/	/	0.523±0.145	0.649±0.211
22.954	2-methylhexadecane	/	0.161±0.005	0.191±0.056	0.226±0.046	0.117±0.015	/	0.095±0
23.226	(z)-7-tetradecen-1-yl acetate	/	/	0.406±0.194	0.889±0.094	0.292±0	0.202±0.011	0.211±0.033
23.547	8-Heptadecene	/	0.528±0.109	0.442±0.050	0.810±0.144	0.740±0.461	0.177±0.020	0.338±0
24.580	Heptadecane	0.275±0.080	1.350±1.005	0.749±0.400	1.754±0.081	0.749±0.151	0.518±0.171	0.708±0.053
24.857	phytane	/	/	/	/	/	0.167±0	0.195±0.014
27.591	3-methylheptadecane	/	/	0.083±0	0.092±0	0.070±0	0.088±0	0.105±0.007
28.293	nonadec-1-ene	0.162±0.029	/	/	/	/	0.161±0.038	0.146±0.012
28.494	octadecane	0.298±0.073	0.226±0.080	0.082±0.013	/	0.160±0	0.311±0.031	0.432±0.017

Note: ‘/’ indicates trace amount or not detected，the data are represented by the mean ± standard deviation (n=3). CE: Color-exposure stage; BS: Blooming stage; IF: Initial flowering stage; HO: Half opening stage; FB: Full blooming stage; ID: Initial decay stage; DE: Decay stage.

Table S2 Analysis of floral volatile components of *Paeonia ostia* ‘Fengdan’ at different flower development stages under field cultivation

Peak Time	Substance Name	CE	BS	IF	HO	FB	ID	DE
2.901	Styrene	0.053±0.002	0.078±0	0.020±0.013	/	/	/	/
2.936	m-Xylene	0.126±0.073	0.133±0.055	0.047±0.016	0.139±0.108	/	0.030±0	/
3.65	3-Hexanol	0.169±0.028	0.145±0.030	0.138±0.130	/	/	/	/
4.299	Heptane,2,2,4,6,6-pentamethyl-	0.215±0.062	/	/	/	/	/	/
4.728	(3Z)-hex-3-en-1-yl acetate	0.173±0.076	/	0.027±0	0.061±0	/	/	/
5.59	(E)-β-ocimene	2.782±2.125	4.319±1.760	5.002±4.383	/	0.665±0.142	0.181±0.118	0.846±0.093
6.671	Undecane	/	/	0.144±0.127	0.300±0.138	0.426±0	/	0.290±0
6.698	Nonanal	0.296±0.053	/	0.051±0.038	/	/	/	/
6.97	Phenylethyl Alcohol	/	/	0.276±0	1.158±0.627	0.811±0	0.040±0	/
7.577	(-)-Myrtenol	0.430±0.364	/	/	/	/	/	/
8.873	Dodecane	0.203±0.057	0.154±0.033	1.464±1.433	/	0.252±0	0.069±0.012	/
8.996	Decanal	0.152±0.039	/	/	/	/	/	/
9.55	3-Carene	0.302±0	0.436±0.151	1.011±1.007	0.672±0.362	0.645±0.134	0.233±0.106	1.397±0
9.599	3-Phenylpropanol	/	0.245±0.048	/	/	/	/	/
10.12	nerol	/	0.679±0.228	0.464±0.427	0.813±0.484	0.898±0.312	0.022±0	/
10.276	2-Propen-1-ol, 3-phenyl-	/	0.309±0.056	0.038±0	/	0.350±0.050	/	/
11.126	Tridecane	0.153±0.027	0.484±0.140	0.689±0.551	0.272±0.123	0.942±0.110	0.411±0.135	1.040±0.347
12.52	(+)-3-Carene	/	0.405±0.200	0.092±0.038	1.708±0	/	0.465±0.185	0.425±0
13.411	Tetradecane	3.584±3.169	0.776±0.298	0.501±0.212	0.194±0.129	1.451±0.090	0.791±0.288	2.566±0.795
13.692	1,3,5-trimethoxybenzene	4.407±2.856	1.807±0.405	0.132±0.038	/	2.110±0.274	0.315±0.240	0.925±0
16.309	Pentadecane	0.778±0.089	2.510±0.788	1.096±0.687	1.529±0.722	2.787±0.118	1.062±0.433	8.969±3.523
16.724	2,4-Di-tert-butylphenol	0.113±0.033	0.362±0.106	0.157±0.078	/	/	/	/
20.067	hexadecane	0.529±0.140	1.391±0.462	0.439±0.084	/	1.505±0.069	0.814±0.283	5.045±1.520
22.741	2-methylhexadecane	0.186±0.043	0.397±0.097	0.116±0.048	/	/	/	/
24.358	Heptadecane	1.170±0.317	2.370±0.336	0.371±0.211	1.230±0.063	1.427±0.110	0.765±0.438	2.031±0.767
28.139	1-Octadecene	0.124±0	0.386±0.035	0.075±0.014	/	/	/	/
28.345	octadecane	0.406±0.150	0.651±0.218	0.186±0.032	/	0.796±0.055	0.405±0.142	/
30.029	nonadec-1-ene	0.095±0.041	/	0.010±0	/	/	/	/

Note: ‘/’ indicates trace amount or not detected，the data are represented by the mean ± standard deviation (n=3). CE: Color-exposure stage; BS: Blooming stage; IF: Initial flowering stage; HO: Half opening stage; FB: Full blooming stage; ID: Initial decay stage; DE: Decay stage.

Table S3 Daily variation analysis of floral volatile components of *Paeonia ostia* ‘Fengdan’ under potted cultivation

Peak Time	Substance Name	6:00-9:00	9:00-12:00	12:00-15:00	15:00-18:00
3.025	Ethylbenzene	0.048±0.007	0.045±0	0.044±0.004	0.083±0.002
3.112	p-Xylene	0.150±0.019	0.151±0.020	0.136±0.018	0.165±0.024
3.406	m-Xylene	0.050±0	/	0.056±0.001	0.131±0.014
4.970	Decane	/	/	/	0.095±0.022
5.897	(E)-β-ocimene	0.698±0.252	0.301±0.155	1.140±0.537	1.150±0.545
6.923	Undecane	0.072±0.011	/	0.045±0	0.294±0.082
7.026	Nonanal	/	/	0.071±0	0.329±0.033
9.075	Dodecane	0.148±0.028	0.081±0	0.083±0.014	0.341±0.171
11.266	Tridecane	0.430±0.228	0.154±0.021	0.201±0.058	0.505±0.222
13.558	Tetradecane	0.485±0.157	0.188±0.024	0.251±0.017	0.497±0.155
13.846	1,3,5-trimethoxybenzene	2.124±0.724	1.193±0.222	2.377±0.865	2.404±0.732
16.480	Pentadecane	1.391±0.452	0.631±0.228	1.201±0.247	1.687±0.900
16.901	2,4-Di-tert-butylphenol	0.390±0.142	0.249±0.028	0.167±0.112	0.354±0.087
20.276	hexadecane	0.883±0.186	0.188±0.002	0.432±0.123	0.964±0.188
22.305	(-)-methyl jasmonate	0.621±0.087	0.142±0	0.509±0	/
22.954	2-methylhexadecane	0.231±0.060	/	0.138±0.017	0.234±0.128
23.226	(z)-7-tetradecen-1-yl acetate	0.684±0.201	0.365±0.169	0.533±0.052	0.669±0.345
23.547	8-Heptadecene	/	0.518±0.361	0.673±0.111	0.837±0.424
24.580	Heptadecane	1.324±0.219	0.944±0.484	2.015±0.134	1.990±1.134
24.857	phytane	/	/	/	0.203±0.020
27.591	3-methylheptadecane	0.348±0.043	/	0.138±0.027	0.217±0.079
28.293	nonadec-1-ene	/	/	/	0.193±0.045
28.494	octadecane	0.360±0.092	/	0.193±0.047	0.364±0.074

Note: ‘/’ indicates trace amount or not detected，the data are represented by the mean ± standard deviation (n=3).

Table S4 Daily variation analysis of floral volatile components of *Paeonia ostia* ‘Fengdan’ under field cultivation

Peak Time	Substance Name	6:00-9:00	9:00-12:00	12:00-15:00	15:00-18:00
2.936	m-Xylene	0.078±0.011	0.036±0.006	0.204±0.020	0.062±0.007
3.650	3-Hexanol	0.016±0	0.052±0.019	0.111±0.039	0.217±0
4.299	Heptane, 2,2,4,6,6-pentamethyl-	0.041±0.005	0.065±0.009	/	0.237±0
5.590	(E)-β-ocimene	0.090±0.028	0.415±0.266	0.660±0.410	5.445±4.916
5.797	Hexane, 3,3-dimethyl-	0.016±0	0.029±0.005	/	0.089±0
6.671	Undecane	0.048±0.025	0.171±0.156	0.078±0.030	0.212±0
8.873	Dodecane	0.037±0.005	0.033±0.001	0.094±0	/
9.550	3-Carene	0.066±0.034	0.151±0.054	0.292±0.112	0.271±0.161
10.120	nerol	0.069±0.031	0.348±0.075	0.559±0.179	0.257±0.128
10.276	2-Propen-1-ol, 3-phenyl-	0.044±0	0.449±0.334	0.181±0	0.166±0.075
11.126	Tridecane	0.088±0.010	0.132±0.038	0.374±0.096	0.943±0.843
13.411	Tetradecane	0.090±0.031	0.127±0.021	1.318±0.462	0.552±0.278
13.692	1,3,5-trimethoxybenzene	0.689±0.309	1.734±0.448	2.317±0.783	3.486±2.845
16.309	Pentadecane	0.474±0.299	0.643±0.269	3.791±1.186	1.919±1.252
16.724	2,4-Di-tert-butylphenol	0.024±0	0.097±0.025	0.171±0	0.167±0
20.067	hexadecane	0.083±0.054	0.116±0.014	2.214±0.795	0.579±0.201
22.741	2-methylhexadecane	0.059±0.010	0.151±0.102	0.322±0.019	0.202±0
24.358	Heptadecane	0.388±0.198	0.811±0.418	1.339±0.397	1.069±0.468
28.345	octadecane	0.068±0.034	0.112±0.022	0.309±0.125	0.234±0.027
29.671	Nonadecane, 2-methyl-	0.025±0.007	0.030±0	/	/
30.029	nonadec-1-ene	0.170±0.055	0.079±0.048	0.594±0	1.377±0
30.116	1-Eicosene	0.041±0	0.145±0.079	0.092±0	0.194±0

Note: ‘/’ indicates trace amount or not detected，the data are represented by the mean ± standard deviation (n=3).
